# Investigation of Statin Medication Use in Elderly Patients with Cardiovascular Disease on Regular Physical Examination and the Relationship with Glucolipid Metabolism and Adverse Cardiovascular Prognosis

**DOI:** 10.1155/2022/8714392

**Published:** 2022-06-16

**Authors:** Chang Liu, Rui Ma, Daifeng Gao, Bin Hu, Xiaolei Yin, Zhipeng Liu, Hu Lin, Zhenhua Zhang

**Affiliations:** ^1^Physical Examination Center, The 305th Hospital of the PLA, Beijing, China; ^2^Department of Pharmacy, The 305th Hospital of the PLA, Beijing, China; ^3^Department of Geriatric Medicine, Health Division of Guard Bureau, Joint Staff of the Central Military Commission, Beijing, China; ^4^Department of Stomatology, The 305th Hospital of the PLA, Beijing, China; ^5^Department of Ophthalmology, The 305th Hospital of the PLA, Beijing, China; ^6^Department of Medical Administration, The 305th Hospital of the PLA, Beijing, China; ^7^Department of Gastroenterology, The 305th Hospital of the PLA, Beijing, China

## Abstract

Our purpose of this study was to investigate the use of statins in elderly patients with cardiovascular diseases during regular physical examination and to analyze the relationship between statins and glucose and lipid metabolism and adverse cardiovascular prognosis. From January 2019 to December 2021, 2121 elderly patients with cardiovascular disease underwent regular physical examination as the study subjects to investigate the use and intensity of statins. The patients were divided into the dosing group (*n* = 1848) and the nondosing group (*n* = 273) according to whether they were taking statins or not. The cardiac function, glucose and lipid metabolism indexes, and cardiovascular adverse events were compared between the two groups. Statin use in elderly patients with cardiovascular disease was 87.13% (1848/2121). The intensity of statin use decreased with age (*P* < 0.05); the left ventricular ejection fraction (LVEF) was greater in the medicated group than in the nonmedicated group, and the left ventricular end-diastolic internal diameter (LVDd) and left ventricular end-systolic internal diameter (LVDs) were smaller than in the nonmedicated group (*P* < 0.05). The total cholesterol (TC), triglyceride (TG), low-density lipoprotein cholesterol (LDL-C), and fasting blood glucose (FBG) levels were lower in the medicated group than in the nonmedicated group, the high-density lipoprotein cholesterol (HDL-C) levels were higher than in the nonmedicated group, and the glycated hemoglobin (HbA1c) values were lower than in the nonmedicated group (*P* < 0.05). The overall incidence of cardiovascular adverse events in the medicated group was lower than that in the nonmedicated group (*P* < 0.05). Statin use was higher in elderly patients with cardiovascular disease; the intensity of drug use decreased with age. The patients' cardiac function, glucose metabolism, and prognosis were significantly improved after statin treatment.

## 1. Introduction

Cardiovascular diseases are caused by atherosclerosis, including coronary heart disease, stroke, and peripheral vascular disease. It is now clinically recognized that disorders of lipid metabolism are an important factor in the development of cardiovascular disease [1]. Statins are commonly used lipid-lowering drugs that not only lower lipid levels but also have the functions of antioxidant, inhibition of cardiac hypertrophy, and inhibition of neuroendocrine hyperactivation [2, 3]. Related reports also point out that statins can improve endothelial cell function, restore autonomic function, and prevent abnormal myocardial perfusion due to microvascular spasm [4]. Most of the current clinical evidence on the benefits of statins and drug safety comes from nonelderly patients, and there are few studies on statin use in elderly patients. Therefore, this study investigated statin medication use in elderly patients with cardiovascular disease through regular physical examination. We also analyzed the relationship between statin use and glucolipid metabolism as well as adverse cardiovascular prognosis. This study will further provide a clinical basis for the use of statins in elderly patients.

## 2. Patients and Methods

### 2.1. Patients

This study was approved by the ethics committee of The 305th Hospital of the PLA. All participants signed written informed consents before the study. 2,121 elderly patients with cardiovascular disease who had regular physical examination from January 2019 to December 2021 were enrolled as the study population. The inclusion criteria were as follows: (i) patients that met the diagnostic criteria of Practical Cardiology on cardiovascular diseases [5]; (ii) patients' age were ≥60 years; and (iii) those with complete clinical data. Exclusion criteria were as follows: (i) patients with combined malignant neoplastic diseases; (ii) patients with combined liver and kidney insufficiency; (iii) patients with alcoholism or history of drug abuse; and (iv) patients with combined infectious diseases.

### 2.2. Methods

Patients were counted on atorvastatin, rosulvastatin, simvastatin, fluvastatin and pitavastatin, and the intensity of use. The intensity of use was based on the relevant guidelines [6], with high intensity being 20 mg of rosuvastatin and 40-80 mg of atorvastatin; medium intensity being 10-<40 mg of atorvastatin, 5-<20 mg of rosuvastatin, 2-4 mg of pitavastatin, 80 mg of fluvastatin, and 20-40 mg of simvastatin; and low intensity being doses below medium intensity.

The hospital information system was used to inquire about the patients' age, gender, smoking history, drinking history, and other general data and past medical history such as hypertension history and diabetes history.

Vivid-7 Doppler color echocardiography of GE company was used to determine left ventricular end dimension (LVDd), left ventricular end systolic dimension (LVDs), and left ventricular ejection fraction (LVEF).

3 mL of fasting venous blood was collected from the patients within 24 hours after enrollment, and the total cholesterol (TC), triglyceride (TG), low-density lipoprotein cholesterol (LDL-C), high-density lipoprotein cholesterol (HDL-C), fasting blood glucose (FBG), and glycosylated hemoglobin A1c (HbA1c) levels were measured using Beckman Coulter DXC800 system.

The incidence of major cardiovascular adverse events such as acute myocardial infarction and heart failure in the two groups was statistically analyzed.

### 2.3. Observation Index

The use of statins and the dose of statins were counted, and the patients were divided into a medicated group and a nonmedicated group according to whether they were taking statins or not. The cardiac function, glucose and lipid metabolism indexes, and the occurrence of cardiovascular adverse events were compared between the two groups.

### 2.4. Statistical Analysis

Statistical Product and Service Solutions (SPSS) 23.0 (IBM, Armonk, NY, USA) was applied for statistical analysis. Independent sample *t*-test was used for comparison between the groups for measurement data obeying normal distribution and was used for comparison within the groups, all expressed as ($\bar{\text{x}}\pm \text{s}$). Count data were tested by *χ*^2^ and expressed as rate (%), *P* < 0.05 indicates statistical difference.

## 3. Results

### 3.1. Statin Use in Elderly Patients with Cardiovascular Disease

The use rate of statins in elderly patients with cardiovascular disease was 87.13% (1848/2121). There was no significant difference in the use of statins in patients of different ages (*P* > 0.05) (Table 1); the intensity of statin use in patients decreased with increasing age (*P* < 0.05) (Table 1).

### 3.2. Clinical Data of Patients in Nonmedicated Group

The patients were divided into medicated group (*n* = 1848) and nonmedicated group (*n* = 273) according to whether they took statins or not. There was no significant difference in clinical data between the two groups (*P* > 0.05) (Table 2).

### 3.3. Cardiac Function Indexes in the Nonmedicated Group

The LVEF value in the medicated group was greater than that in the nonmedicated group, and the LVDs and LVDd values were less than those in the nonmedicated group (*P* < 0.05) (Table 3).

### 3.4. Glucose and Lipid Metabolism Indexes in the Nonmedicated Group

The levels of TG, TC, LDL-C, and FBG in the medicated group were lower than those in the nonmedicated group, the level of HDL-C was higher than that in the nonmedicated group, and the HbA1c value was lower than that in the nonmedicated group (*P* < 0.05) (Table 4).

### 3.5. The Incidence of Cardiovascular Adverse Events in the Nonmedicated Group

The total incidence of cardiovascular adverse events in the medicated group was lower than that in the nonmedicated group (*P* < 0.05) (Table 5).

## 4. Discussion

At present, it is believed that improving lipid metabolism disorders is the key to the treatment of cardiovascular diseases. Statins can inhibit cholesterol synthase to play a lipid-lowering role and are widely used in cardiovascular diseases. Decreased function of various organs and changes in statin metabolism in elderly patients have led to few clinical reports on statin use in elderly patients. In this study, we analyzed the use of statins in elderly patients and found that the use of other statins was 87.13%, which was slightly higher than that in the study [7]. This may be related to the higher treatment compliance of patients with regular physical examinations. Clinical data showed that the lipid-lowering effect of statins was closely related to drug dose [8], but relevant studies have pointed out that high-dose statins have more adverse reactions in the treatment of cardiovascular diseases, while the patient's age is too high and physical function is also weaker [9], so the dose needs to be adjusted according to the patient's age. The results of this study showed that the intensity of statin use in patients decreased with increasing age.

Statins can significantly reduce plasma cholesterol concentration, delay and inhibit the progression of atherosclerosis, and have become one of the drugs of choice for lipid-lowering therapy and antiatherosclerosis in clinical practice [10]. The results of this study showed that the TC, TG, and LDL-C levels in the medicated group were higher than those in the unmedicated group, and the HDL-C level was higher than that in the unmedicated group, indicating that statin treatment for elderly patients with cardiovascular disease can reduce the body's blood lipid level, which is mainly because statins can play a role in regulating blood lipids by competitively inhibiting enzyme secretion in hepatocytes and reducing the body's methyl dehydroabietate content [11]. In this study, we found that FBG levels were lower and HbA1c values were lower in the medicated group than in the unmedicated group, indicating that statins can improve glucose metabolism in elderly patients with cardiovascular disease, which is mainly related to the fact that statins can improve insulin resistance and glucose tolerance through their effects.

Myocardial function in patients with cardiovascular disease is affected by the amount of cholesterol in the cardiac muscle cells. Increased cholesterol levels in patients can lead to reduced cell membrane function, which in turn can affect myocardial function in patients. Statins can improve cardiac function because they reduce body cholesterol levels [12]. Relevant studies have pointed out that statins can improve glucose and lipid metabolism, reduce hyperglycemia-related sclerosing stress, and improve endothelial function [13]. It has also been reported that statins, in addition to their lipid-lowering effects, can also reduce platelet adhesiveness, prevent thrombosis, and improve endothelial function [14]. Statins can downregulate angiotensin II receptor content and reduce the degree of myocardial fibrosis, while improving myocardial function in patients [15]. The results of this study showed that the LVEF value of the medicated group was greater than that of the unmedicated group, and LVDd and LVDs were less than those of the unmedicated group, indicating that statins in the treatment of cardiovascular disease can improve the patient's cardiac function, which is mainly because statins can reduce the body cholesterol content, reduce the amount of cholesterol in the cell membrane, and then play a role in improving the patient's myocardial cell function. Some scholars have found that statins have the effect of stabilizing plaques, reducing the adhesion and aggregation of inflammatory cells and platelets in the microcirculation and preventing the activation of the coagulation system, which can improve myocardial blood perfusion in patients [16]. The results of this study showed that the total incidence of cardiovascular adverse events in the medicated group was lower than that in the unmedicated group, indicating that statin therapy given to patients with cardiovascular disease can reduce cardiovascular adverse events. The reason for this is that statins can improve body hemodynamics and promote apoptosis and fibrosis of cardiomyocytes and interstitium by exerting selective inhibitory effects, inhibiting the activity of reductase, the rate-limiting enzyme of synthesis, and reducing intracellular cholesterol synthesis in the human body, which can reduce the incidence of cardiovascular adverse events [17]. This study has the following advantages over other previous related studies: (1) larger sample size; (2) higher proportion of elderly patients; and (3) more detailed observation indicators.

## 5. Conclusion

Statin use is higher in elderly patients with cardiovascular disease, and the intensity of drug use decreases with increasing age. Patients' cardiac function, glucose metabolism indicators, and prognosis are significantly improved after statin treatment.

## Figures and Tables

**Table 1:** Analysis of statin use in elderly patients with cardiovascular disease.

Item	60-69 years (*n* = 795)	70-79 years (*n* = 637)	≥80 years (*n* = 689)
Type of drug			
Atorvastatin calcium	330 (41.51)	260 (40.82)	285 (41.36)
Rosuvastatin calcium	322 (40.50)	254 (39.87)	276 (40.06)
Simvastatin	33 (4.15)	29 (4.55)	28 (4.06)
Fluvastatin	9 (1.13)	7 (1.10)	6 (0.87)
Pitavastatin	4 (0.5)	3 (0.47)	2 (0.29)
Drug intensity			
Low-intensity statin	171 (21.51)	214 (33.59)	543 (78.81)
Moderate-intensity statin	525 (66.04)	337 (52.90)	54 (7.84)
High-intensity statin	2 (0.25)	2 (0.31)	0 (0.00)
Total	698 (87.80)	553 (86.81)	597 (86.65)

Note: Comparison of statin intensity among the three groups, *P* < 0.05.

**Table 2:** Comparison of clinical data of medicated group.

Classification	Administration group (*n* = 1848)	Unmedicated group (*n* = 273)	*Χ* ^2^/*t*	*P*
Age (years)	73.65 ± 6.09	73.52 ± 6.58	0.326	0.745
Gender: male (case)	1168 (63.20)	159 (58.24)	2.500	0.114
BMI (body mass index) (kg/m^2^)	22.34 ± 2.06	22.19 ± 2.24	1.110	0.267
Complicated with hypertension (*n*)	1083 (58.60)	147 (53.85)	2.120	0.137
Complicated with hyperlipidemia (case)	629 (34.04)	78 (28.57)	3.197	0.074
Complicated with diabetes (case)	417 (22.56)	53 (19.41)	1.369	0.242
Smoking history (case)	563 (30.47)	97 (35.53)	2.848	0.092
Alcohol history (case)	438 (23.70)	57 (20.88)	1.059	0.303

**Table 3:** Comparison of cardiac function indicators in the medicated group.

Group	*N*	LVEF (%)	LVDs (mm)	LVDd (mm)
Administration group	1848	51.09 ± 3.52	47.56 ± 5.07	61.38 ± 6.25
Nonmedicated group	273	50.63 ± 3.14	48.27 ± 5.94	62.71 ± 6.09
*t*		2.042	2.110	3.293
*P*		0.041	0.035	0.001

**Table 4:** Comparison of glucose and lipid metabolism indicators in the medicated group.

Group	*N*	TG (mmol/L)	TC (mmol/L)	LDL-C (mmol/L)	HDL-C (mmol/L)	FBG (mmol/L)	HbA1c (%)
Administration group	1848	1.92 ± 0.21	4.79 ± 0.73	2.95 ± 0.41	1.34 ± 0.24	5.79 ± 1.12	6.08 ± 1.24
Nonmedicated group	273	2.05 ± 0.23	5.17 ± 0.69	3.11 ± 0.67	1.29 ± 0.23	6.08 ± 1.15	6.33 ± 1.36
*t*		9.427	8.084	5.462	3.230	3.980	3.070
*P*		<0.001	<0.001	<0.001	0.001	<0.001	0.002

**Table 5:** Comparison of incidence of cardiovascular adverse events in the nonmedicated group (case, %).

Group	*N*	Acute myocardial infarction	Heart failure	Cardiovascular death	Revascularization	Total occurrence
Administration group	1848	46	21	10	17	5.09 (94)
Nonmedicated group	273	11	8	3	4	9.52 (26)
*Χ* ^2^						8.774
*P*						0.003

## Data Availability

The datasets used and analyzed during the current study are available from the corresponding author on reasonable request.

## References

[1] Chamberlain A. M., Cohen S. S., Killian J. M., Monda K. L., Weston S. A., Okerson T. (2019). Lipid-Lowering prescription patterns in patients with diabetes mellitus or cardiovascular disease. *The American Journal of Cardiology*.

[2] Nicholls S. J., Puri R., Anderson T. (2016). Effect of evolocumab on progression of coronary disease in statin-treated patients. *JAMA*.

[3] Li S., Schooling C. M. (2022). Investigating the effects of statins on ischemic heart disease allowing for effects on body mass index: a Mendelian randomization study. *Scientific Reports*.

[4] Li X., Xiao H., Lin C. (2019). Synergistic effects of liposomes encapsulating atorvastatin calcium and curcumin and targeting dysfunctional endothelial cells in reducing atherosclerosis. *International Journal of Nanomedicine*.

[5] Lu H., Roux O., Fournier S. (2022). Cardiologie. *Revue Médicale Suisse*.

[6] Opoku S., Gan Y., Yobo E. A. (2021). Awareness, treatment, control, and determinants of dyslipidemia among adults in China. *Scientific Reports*.

[7] Strandberg T. E. (2019). Role of statin therapy in primary prevention of cardiovascular disease in elderly patients. *Current Atherosclerosis Reports*.

[8] Horodinschi R. N., Stanescu A., Bratu O. G., Pantea S. A., Radavoi D. G., Diaconu C. C. (2019). Treatment with statins in elderly patients. *Medicina*.

[9] Zhang Q. H., Yin R. X., Chen W. X., Cao X. L., Chen Y. M. (2018). Association between the TIMD4-HAVCR1 variants and serum lipid levels, coronary heart disease and ischemic stroke risk and atorvastatin lipid-lowering efficacy. *Bioscience Reports*.

[10] Xing Y. Y., Liu J., Liu J. (2019). Statin use and low-density lipoprotein cholesterol levels in patients aged 75 years and older with acute coronary syndrome in China. *Zhonghua Xin Xue Guan Bing Za Zhi*.

[11] Jamialahmadi T., Baratzadeh F., Reiner Z. (2021). The effects of statin dose, lipophilicity, and combination of statins plus ezetimibe on circulating oxidized low-density lipoprotein levels: a systematic review and meta-analysis of randomized controlled trials. *Mediators of Inflammation*.

[12] De La Cruz J. A., Mihos C. G., Horvath S. A., Santana O. (2019). The pleiotropic effects of statins in endocrine disorders. *Endocrine, Metabolic & Immune Disorders Drug Targets*.

[13] Tousoulis D., Antoniades C., Vassiliadou C. (2005). Effects of combined administration of low dose atorvastatin and vitamin E on inflammatory markers and endothelial function in patients with heart failure. *European Journal of Heart Failure*.

[14] Hirota T., Fujita Y., Ieiri I. (2020). An updated review of pharmacokinetic drug interactions and pharmacogenetics of statins. *Expert Opinion on Drug Metabolism & Toxicology*.

[15] Kazi D. S., Penko J. M., Bibbins-Domingo K. (2017). Statins for primary prevention of cardiovascular disease: review of evidence and recommendations for clinical practice. *The Medical Clinics of North America*.

[16] Xie C., Zhu M., Hu Y., Wang K. (2020). Effect of intensive and standard lipid-lowering therapy on the progression of stroke in patients with coronary artery syndromes: a meta-analysis of randomized controlled trials. *Journal of Cardiovascular Pharmacology*.

[17] van Stee M. F., de Graaf A. A., Groen A. K. (2018). Actions of metformin and statins on lipid and glucose metabolism and possible benefit of combination therapy. *Cardiovascular Diabetology*.

